# High-fat diet and glucocorticoid treatment cause hyperglycemia associated with adiponectin receptor alterations

**DOI:** 10.1186/1476-511X-10-11

**Published:** 2011-01-18

**Authors:** Cristiane de Oliveira, Ana BM de Mattos, Carolina Biz, Lila M Oyama, Eliane B Ribeiro, Cláudia Maria Oller do Nascimento

**Affiliations:** 1 Disciplina de Fisiologia da Nutrição, Departamento de Fisiologia, Universidade Federal de São Paulo, São Paulo, Brasil

## Abstract

**Background:**

Adiponectin is the most abundant plasma protein synthesized for the most part in adipose tissue, and it is an insulin-sensitive hormone, playing a central role in glucose and lipid metabolism. In addition, it increases fatty acid oxidation in the muscle and potentiates insulin inhibition of hepatic gluconeogenesis. Two adiponectin receptors have been identified: AdipoR1 is the major receptor expressed in skeletal muscle, whereas AdipoR2 is mainly expressed in liver. Consumption of high levels of dietary fat is thought to be a major factor in the promotion of obesity and insulin resistance. Excessive levels of cortisol are characterized by the symptoms of abdominal obesity, hypertension, glucose intolerance or diabetes and dyslipidemia; of note, all of these features are shared by the condition of insulin resistance. Although it has been shown that glucocorticoids inhibit adiponectin expression in vitro and in vivo, little is known about the regulation of adiponectin receptors. The link between glucocorticoids and insulin resistance may involve the adiponectin receptors and adrenalectomy might play a role not only in regulate expression and secretion of adiponectin, as well regulate the respective receptors in several tissues.

**Results:**

Feeding of a high-fat diet increased serum glucose levels and decreased adiponectin and adipoR2 mRNA expression in subcutaneous and retroperitoneal adipose tissues, respectively. Moreover, it increased both adipoR1 and adipoR2 mRNA levels in muscle and adipoR2 protein levels in liver. Adrenalectomy combined with the synthetic glucocorticoid dexamethasone treatment resulted in increased glucose and insulin levels, decreased serum adiponectin levels, reduced adiponectin mRNA in epididymal adipose tissue, reduction of adipoR2 mRNA by 7-fold in muscle and reduced adipoR1 and adipoR2 protein levels in muscle. Adrenalectomy alone increased adiponectin mRNA expression 3-fold in subcutaneous adipose tissue and reduced adipoR2 mRNA expression 2-fold in liver.

**Conclusion:**

Hyperglycemia as a result of a high-fat diet is associated with an increase in the expression of the adiponectin receptors in muscle. An excess of glucocorticoids, rather than their absence, increase glucose and insulin and decrease adiponectin levels.

## Background

Adiponectin, the most abundant plasma protein that is synthesized from differentiated adipocytes, has reduced plasma levels in clinical conditions associated with insulin resistance, including obesity, type 2 diabetes, dyslipidemia and hypertension [1,2]. Furthermore, adiponectin levels are inversely associated with visceral adiposity [3].

Several studies have demonstrated that adiponectin has a central role in glucose and lipid metabolism. Accordingly, the infusion of adiponectin in mice decreased the expression of hepatic gluconeogenesis enzymes, inhibited glucose production and increased the hepatic effect of insulin [4]. In addition to its effect on glucose levels, adiponectin improved insulin sensitivity by reducing the levels of free fatty acids in the plasma and by increasing their oxidation in the muscle, according to some authors [5,6]. Moreover, adiponectin has been reported to exhibit anti-atherosclerotic and anti-inflammatory effects [7,8].

Two adiponectin receptors have been identified: adipoR1 is the major receptor expressed in skeletal muscle, whereas adipoR2 is mainly expressed in liver [9-11]. These receptors are also expressed in adipose tissue (with adipoR1 being expressed 10 to15-fold higher than adipoR2), macrophages and pancreatic beta cells [11-13].

A study by Rasmussen et al [13] found that weight loss caused upregulation of the gene expression of the adiponectin receptors in human adipose tissue. Additionally, it has been demonstrated that there is a weight loss-induced improvement in insulin sensitivity that could be mediated by the upregulation of adiponectin [14].

It has been previously demonstrated that saturated fatty acids increase insulin resistance and the incidence of cardiovascular disease and that monounsaturated fatty acids (MUFA) and polyunsaturated fatty acids (PUFA) are protective against the development of these diseases [15,16]. Few studies have investigated the effect of diet composition on the expression of adiponectin and its receptors. One such study showed that that a high calorie diet decreased serum adiponectin levels [17]. Bullen et al [18] showed that age and a high-fat diet, both of which predispose an organism to obesity and insulin resistance, reduced adiponectin and increased adipoR1 and adipoR2 levels. Furthermore, Mullen et al [19] found that 3 days of feeding a diet high in saturated fat induced adiponectin resistance in the soleus muscle of rats; this was not observed when the rats were treated with a diet high in PUFA. Thus, the authors concluded that the type of fatty acids in the diet is a critical factor in the development of adiponectin resistance.

We have previously demonstrated that feeding a diet rich in saturated fat and PUFA for 2 days resulted in decreased serum adiponectin concentrations and adiponectin gene expression in the retroperitoneal adipose tissue of mice. Similar results were observed after 8 weeks of a PUFA-rich diet treatment only [20]. Additionally, the 8-week treatment with a PUFA-rich diet increased the corticosterone plasma concentrations in rats [21].

An excess of cortisol, as seen in Cushing's syndrome or with clinical administration of glucocorticoids that is used to treat acute and chronic inflammatory diseases, leads to symptoms of abdominal obesity, hypertension, glucose intolerance or diabetes and dyslipidemia, all of which are also features of insulin resistance [22-24]. The decrease of glucocorticoids with an adrenalectomy can reverse hyperglycemia in many models of obesity [25-27] and can increase insulin sensitivity in obese mice [28].

Previous studies have shown that glucocorticoids inhibit adiponectin expression *in vitro *and in animal models [29-32] and that adrenalectomy can stimulate adiponectin expression in the white adipose tissue of ob/ob mice [33]. However, few studies have analyzed the effects of glucocorticoids on the expression of adiponectin receptors, and the results of these studies have been controversial. In hepatocytes, dexamethasone stimulated the adipoR2 promoter, and this effect was abolished by the glucocorticoid receptor antagonist RU486 [34]. In contrast, dexamethasone decreased adipoR2 mRNA expression in human skeletal muscle [32].

The aims of the present study were to investigate the effects of a high-fat diet on adiponectin receptor expression and to determine the effects of adrenalectomy and dexamethasone treatment in rats treated with a high-fat diet on the expression levels of adiponectin and adiponectin receptors in adipose tissue, skeletal muscle and liver.

## Materials and methods

### Animals and diets

Eight-week-old male Wistar rats were obtained from the Universidade Federal de São Paulo, Centro de Desenvolvimento de Modelos Experimentais (CEDEME) and housed under controlled conditions with a 12-h light/dark cycle (lights on at 6 am) at 22 ± 1°C in the animal room of the Division of Nutrition Physiology with free access to food and water. All animal procedures were conducted in accordance with the guidelines for the care and use of laboratory animals and were approved by the Committee on Animal Research Ethics of the Federal University of São Paulo (Process n° 01201-6).

At 9 weeks of age, rats were fed a normal chow diet (C; 5% fat) or a high-fat diet (HF; control diet enriched with 15% soybean oil) for 21 days.

The HF diet was prepared in the Laboratory of Nutrition Physiology using the commercial chow Nuvilab CR1 (Paraná, Brazil) with casein added to obtain a final 20% protein content and enrichment with 15% (w/w) of commercial soybean oil (Liza, Brazil) and 0.013% butylated hydroxytoluene (w/w). Water was added to obtain the consistency necessary to allow homogenization of the mixture. After homogenization, the mixture was passed through a milling machine to produce pellets that were then dried in a forced ventilation oven at 60°C for 24 h. The prepared pellets were stored in plastic containers at 4°C.

The HF diet used in this study contained 19.4 g lipid/100 g and 379.0 Kcal/100 g, whereas the control diet contained 3.0 g lipid/100 g and 280.6 Kcal/100 g. The protein content was similar in both diets (± 21.8 g protein/100 g). The fatty acid composition of the diets measured as a percentage of the total lipid content was previously described [20].

### Surgery and experimental procedures

At 12 weeks of age, rats were anesthetized using a mixture of ketamine and xylosine (66.6 and 13.3 mg/Kg, respectively) administered by intraperitoneal injection and then they underwent complete bilateral adrenal gland removal via dorsal incision. Subsequently, rats received dexamethasone reposition (A-DEXA; 0.2 mg/100 g body weight (BW) twice per day) by subcutaneous injections or not (ADREC). A total dosage of 0.4 mg per 100 g BW per day was used to guarantee treatment with a supra-physiological dose. Sham adrenalectomized rats (S-ADREC) underwent a similar surgery without removal of the adrenal glands and received subcutaneous injection of 0.9% saline solution twice daily. Saline solution was provided as an additional fluid to adrenalectomized rats. The animals were sacrificed by decapitation without sedation 72 h after surgery. Retroperitoneal (RET), epididymal (EPI) and subcutaneous (SUB) adipose tissues, as well as the gastrocnemius muscle (MUSC) and liver were collected, frozen in liquid nitrogen and stored at -80°C for RNA and protein extraction. Trunk blood was collected and immediately centrifuged; the resulting serum was stored at -80°C until the measurement of serum glucose, insulin, corticosterone and adiponectin concentrations.

### Biochemical and hormonal serum analysis

Commercial ELISA kits were used to measure the serum levels of adiponectin (ALPCO and AdipoGen) and insulin (LINCO and Millipore). Blood glucose levels were determined using the Glucose PAP Liquiform kit (Labtest) and serum corticosterone was assayed by Enzyme Immunoassay (EIA) (Cayman Chemical Co).

### Carcass lipid and protein contents

Carcasses were eviscerated, weighed, and stored at -20°C. The lipid content was measured [35] and standardized [36] as previously described. Briefly, the eviscerated carcasses were autoclaved at 120°C for 90 min and homogenized with a double body weight of water. Aliquots of this homogenate were taken in triplicate, weighed and digested in 3 ml 30% KOH and 3 ml ethanol for at least 2 h at 70°C in capped tubes. After cooling, 2.5 ml 6N H_2_SO_4 _was added, and the samples were washed 3 times with petroleum ether for lipid extraction. Results are expressed as grams of lipid per 100 g of carcass. For protein measurements, aliquots of the same homogenate (weighing approximately 1 g) were heated to 37°C for 1 h in 0.6 N KOH with constant shaking. After clarification by centrifugation, protein content was measured according to a previously described method [37].

### RNA extraction and complementary DNA synthesis

Total RNA was isolated from adipose tissue, muscle and liver samples using TRIzol (Invitrogen) according to the manufacturer's recommendations. The concentrations of RNA samples were determined by measuring the absorbance at 260 nm. The purity of the RNA samples was determined by calculating the absorbance ratios between 260 and 280 nm and by electrophoresis using an ethidium bromide-stained gel. For reverse transcription, 1 μg of total RNA was reverse transcribed using an M-MLV reverse transcriptase kit (Promega) and the following reaction conditions: 65°C for 10 min, 37°C for 60 min and 65°C for 10 min.

### Real-time PCR

The relative levels of adiponectin, adipoR1 and adipoR2 mRNAs were measured in duplicate by real-time PCR using SYBR green dye and an ABI Prism 7300 sequence detection system (Applied Biosystems, USA). Reactions were incubated as follows: an initial denaturation step at 95°C for 10 min followed by 40 cycles of 95°C for 15 s and 60°C for 1 min and culminating with melt curve analysis. All results were normalized to the values of the housekeeping gene hypoxanthine phosphoribosyltransferase (HPRT) and expressed as the fold change relative to the control group using the 2-ΔΔCt method [38]. The following rat primer sets were used: adiponectin forward, 5'-AATCCTGCCCAGTCATGAAG, and reverse, 5'-CATCTCCTGGGTCACCCTTA; adipoR1 forward, 5'-CTTCTACTGCTCCCCACAGC, and reverse, 5'-TCCCAGGAACACTCCTGCTC; adipoR2 forward, 5'-ATGTTTGCCACCCCTCAGTA, and reverse, 5'-CAGATGTCACATTTGGCAGG; and HPRT forward, 5'-CTCATGGACTGATTATGGACAGGAC, and reverse, 5'-GCAGG TCAGCAAAGAACTTATAGCC.

### Protein analysis by western blotting

Samples of each collected tissue (0.05-0.3 g) were homogenized in 1.0 mL extraction buffer (100 mM Trizma, pH 7.5, 10 mM EDTA, 1% SDS, 10 mM sodium pyrophosphate, 100 mM sodium fluoride, 10 mM sodium orthovanadate, 2 mM PMSF and 0.25 mg aprotinin protease inhibitor). The lysates were incubated with 10% Triton X-100 for 30 min and then centrifuged at 14,000 rpm for 30 min at 4°C. The supernatant was collected, and protein concentration was determined using a Bradford assay (Bio-Rad, Hercules, California) using bovine serum albumin (BSA) as a reference standard.

The protein samples were treated with Laemmli sample buffer and boiled for 5 min before loading 30-60 μg protein onto a 12% SDS-PAGE gel in a Bio-Rad miniature slab gel apparatus. Electrotransfer of the proteins from the gel to a nitrocellulose membrane was performed for 1 h at 120 V (constant voltage) using a Bio-Rad miniature transfer apparatus. Nonspecific protein binding to the nitrocellulose was reduced by a 2-h preincubation in blocking buffer (1% BSA, 10 mM Tris, 150 mM NaCl and 0.02% Tween 20). The nitrocellulose membranes were incubated overnight at 4°C with antibodies against adipoR1, adipoR2 (both 1:1,000; Santa Cruz Biotechnology, CA, USA) or α-tubulin (1:3,000; Santa Cruz) that were diluted in blocking buffer combined with 1% bovine agarose and then washed for 30 min in blocking buffer without BSA. The membranes were then incubated with secondary antibody, either anti-goat for adipoR1 and adipoR2 (1:10,000) or anti-mouse for α-tubulin (1:5,000), from Santa Cruz Biotechnology. The blots were subsequently incubated with a peroxidase-conjugated secondary antibody for 1 h at 22°C, processed for enhanced chemiluminescence (Amersham ECL Detection Reagent, GE Healthcare) to visualize the immunoreactive bands and developed using Hybond ECL Nitrocellulose membrane (Amersham, GE Healthcare). The protein bands were identified according to their migration rate in comparison to the rainbow recombinant protein molecular weight markers and quantified by densitometry using Image J software. Results are expressed as arbitrary units relative to α-tubulin.

### Statistical analysis

Data are presented as the mean ± SEM. An unpaired Student's t-test was used to assess potential differences between the C and HF diet groups. A one-way ANOVA followed by the Tukey post hoc test were used to compare the results between surgical groups (S-ADREC, ADREC and A-DEXA). Differences were considered to be significant when p < 0.05.

## Results

### 1. The effects of the PUFA-rich diet

The daily energy intakes were similar between the C and HF groups over the 21 days of treatment. The body weight gain of these groups was also similar, with no significant difference in body weight at the end of the feeding experiment (336.60 ± 7.27 g vs. 353.28 ± 5.49 g for the C and HF groups, respectively, p = 0.08). In addition, no significant differences were observed in carcass protein or lipid content or concentrations of serum corticosterone, insulin or adiponectin between the C and HF groups. However, HF diet caused an increase in serum glucose concentrations compared to the control diet (152.16 ± 13.33 vs. 97.72 ± 16.12 mg/dL for HF and C groups, respectively, *p *< 0.01) (Table 1).

**Table 1:** Effects of 21 days of chow diet and high-fat diet

	C group	HF group
**Body weight gain (g)**	82.53 ± 6.7 (15)	77.32 ± 3.51 (25)
**Daily energy intake (Kj)**	5764,72 (15)	5642,22 (25)
**Carcass protein (g/100 g)**	41.61 ± 2.44 (8)	42.31 ± 2.42 (7)
**Carcass lipid (g/100 g)**	10.07 ± 0.58 (12)	11.77 ± 1.08 (10)
**Corticosterone (μg/dL)**	0.637 ± 0.22 (6)	0.526 ± 0.19 (6)
**Glucose (mg/dL)**	97.72 ± 16.12 (8)	152.16 ±13.33 * (6)
**Insulin (ng/mL)**	1.72 ± 0.27 (8)	1.83 ± 0.10 (7)
**Adiponectin (μg/mL)**	10.42 ± 1.07 (6)	12.62 ± 1.21 (7)

HF: high fat diet. Results presented as the mean ± SEM. A Student's t-test was used for statistical comparisons between the HF and control (C) groups; p < 0.05

**Figure 1:**
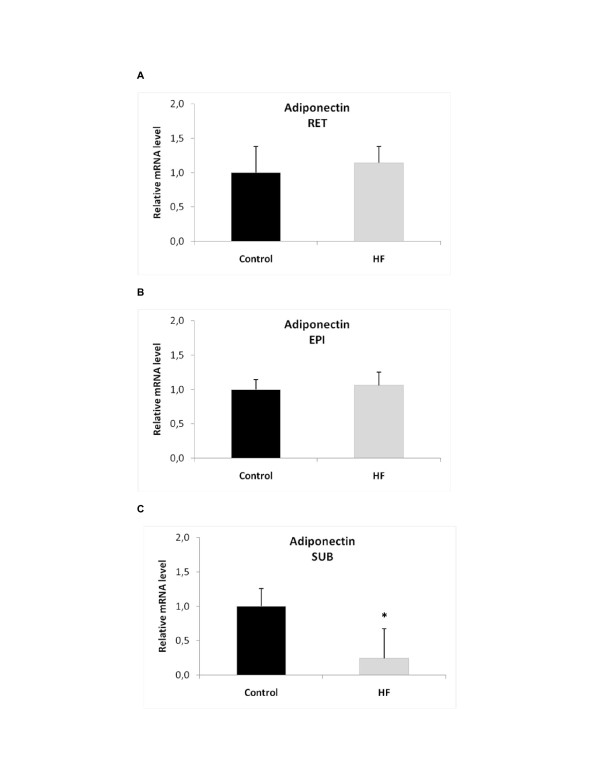
**Effect of a high-fat (HF) diet on adiponectin gene expression in (A) retroperitoneal (RET), (B) epididymal (EPI) and (C) subcutaneous (SUB) adipose tissue**. Fold change data are expressed as the mean ± SEM. Values were normalized to HPRT gene expression with n = 5-9 per group. A Student's t-test was used for statistical comparisons between the HF and control (C) groups; *p < 0.005.

**Figure 2:**
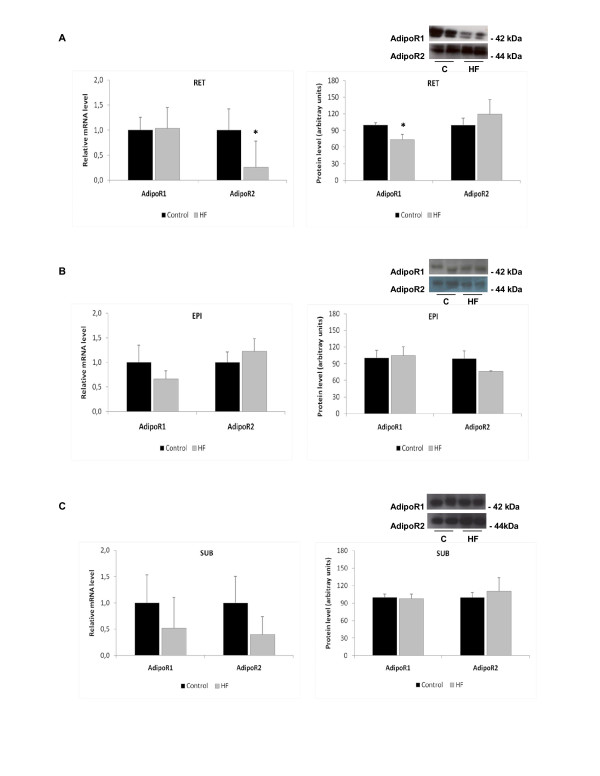
**Effect of a high-fat diet (HF) on the mRNA and protein expression of the adiponectin receptors adipoR1 and adipoR2 in (A) retroperitoneal (RET), (B) epididymal (EPI) and (C) subcutaneous adipose tissue (SUB). Data are expressed as the mean ± SEM. mRNA values were normalized to HPRT, and protein values were normalized to α-tubulin with n = 5-9 per group. **A Student's t-test was used for statistical comparisons between the HF and control (C) groups; *p < 0.05.

Analysis of adiponectin gene expression in various adipose tissue depots showed 4-fold decreased expression in the SUB depot (p < 0.005) of the HF group, but no change in expression in the RET or EPI depots compared to the control group (Figure 1). AdipoR2 gene expression was 4-fold lower in the RET adipose tissue of the HF group compared to the control group (p < 0.05), whereas there was no change in adipoR2 protein content. We did not find any significant alterations in the mRNA or protein levels in the EPI or SUB adipose tissue depots (Figure 2).

**Figure 3:**
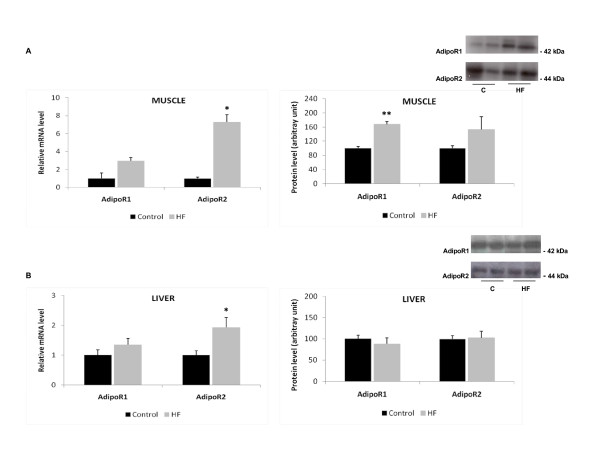
**Effect of a high-fat diet (HF) on the mRNA and protein expression of the adiponectin receptors adipoR1 and adipoR2 in (A) liver and (B) muscle**. Data are expressed as the mean ± SEM. Values were normalized to HPRT with n = 5-9 per group. A Student's t-test was used for statistical comparisons between the HF and control (C) groups; *p < 0.05.

We observed a tissue-specific effect of HF diet on adipoR1 and adipoR2 expression (Figure 3): hepatic adipoR2 gene expression was increased approximately 2-fold (p ≤ 0.05), whereas there was no significant effect on adipoR1 expression and no alteration in the protein levels of either receptor. In the muscle, only adipoR2 gene expression was significantly increased (7-fold increase in HF group compared to controls, p < 0.05); in contrast, there was no significant effect on adipoR1 gene expression, but there was a slight increase in adipoR1 protein expression (67%).

### 2. The effects of corticosterone

As expected, removal of the adrenal glands resulted in reduced serum corticosterone levels. Adrenalectomy did not change glucose or insulin levels. However, in the group that was adrenalectomized and that received dexamethasone treatment (A-DEXA), the serum levels of glucose and insulin were significantly higher than in the sham adrenalectomized (S-ADREC) or adrenalectomized (ADREC) groups (glucose: 310.70 ± 31.70, 152.16 ±13.33 and 144.08 ± 21.25 mg/dL in the A-DEXA, S-ADREC and ADREC groups, respectively; insulin: 5.10 ± 0.81 vs. 1.83 ± 0.10 vs. 1.55 ± 0.37 ng/mL in the A-DEXA, S-ADREC and ADREC groups, respectively). In addition, serum adiponectin levels did not change in the ADREC group, but the A-DEXA group exhibited decreased adiponectin levels (7.01 ± 0.56, 12.62 ± 1.21 and 10.50 ± 1.9 μg/mL in the A-DEXA, S-ADREC and ADREC groups, respectively), showing that an excess of glucocorticoids affects glucose, insulin and adiponectin levels, whereas absence of glucocorticoids does not (Table 2).

**Table 2:** Effects of adrenalectomy and dexamethasone treatment in rats treated with high-fat diet

		HF group		
	**n**	**S-ADREC**	**ADREC**	**A-DEXA**
	
**Corticosterone (μg/dL)**	5-6	0.526 ± 0.19	0.013 ± 0.01^**a**^	0.018 ± 0.01^**a**^
**Glucose (mg/dL)**	5-6	152.16 ±13.33	144.08 ± 21.25	310.70 ± 31.70^**ab**^
**Insulin (ng/mL)**	6-7	1.83 ± 0.10	1.55 ± 0.37	5.10 ± 0.81^**ab**^
**Adiponectin (μg/mL)**	6-7	12.62 ± 1.21	10.50 ± 1.91	7.01 ± 0.56^**a**^

Results presented as the mean ± SEM. A one-way ANOVA followed by a Tukey post-hoc test was used to determine significant differences between groups; *p < 0.05 (effect due to surgery); a ≠ S-ADREC; b ≠ ADREC. Shaw adrenalectomy (S-ADREC), Adrenalectomized group (ADREC), adrenalectomy with dexamethasone treatment (A-DEXA; 0.2 mg/100 g, twice per day).

**Figure 4:**
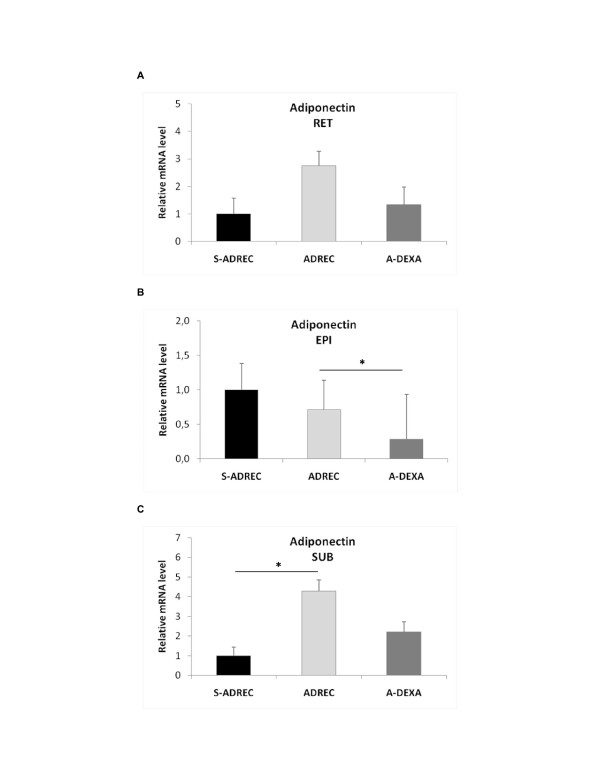
**Effects of adrenalectomy (ADREC) and adrenalectomy with dexamethasone treatment (A-DEXA; 0.2 mg/100 g, twice per day) on adiponectin gene expression in (A) retroperitoneal (RET), (B) epididymal (EPI) and (C) subcutaneous (SUB) adipose tissue in rats treated with HF diet**. Data are expressed as the mean ± SEM. Values were normalized to HPRT levels with n = 5-9 per group. A one-way ANOVA followed by a Tukey post-hoc test was used to determine significant differences between groups (*p < 0.05, effect due to surgery). S-ADREC, sham adrenalectomy.

The adrenalectomy (ADREC) induced a 4-fold increase in adiponectin gene expression of the SUB adipose tissue depot compared to the sham adrenalectomized (S-ADREC) group, but there was no change in that of the RET or EPI depots. In contrast, the A-DEXA group had a 3.5-fold decrease in the mRNA abundance of adiponectin in the EPI adipose tissue compared to the ADREC group (Figure 4).

**Figure 5:**
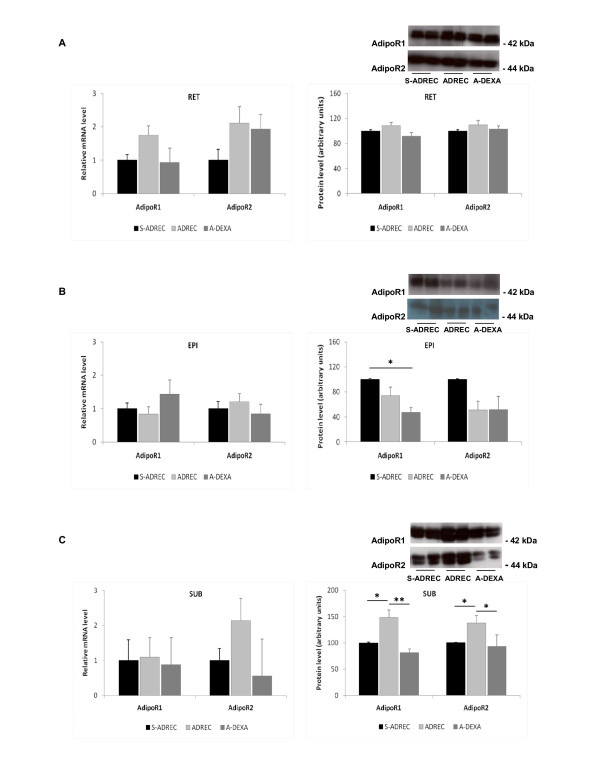
**Effects of adrenalectomy (ADREC) and adrenalectomy with dexamethasone treatment (A-DEXA; 0.2 mg/100 g, twice per day) on the gene and protein expression of the adiponectin receptors adipoR1 and adipoR2 in (A) retroperitoneal (RET), (B) epididymal (EPI) and (C) subcutaneous (SUB) adipose tissue in rats treated with high-fat (HF) diet**. Data are expressed as the mean ± SEM. mRNA values were normalized to HPRT, and protein values were normalized to α-tubulin with n = 5-9 per group. A one-way ANOVA followed by a Tukey post-hoc test was used to determine significant differences between groups (*p < 0.05, **p < 0.01, effect due to surgery). S-ADREC, sham adrenalectomy.

**Figure 6:**
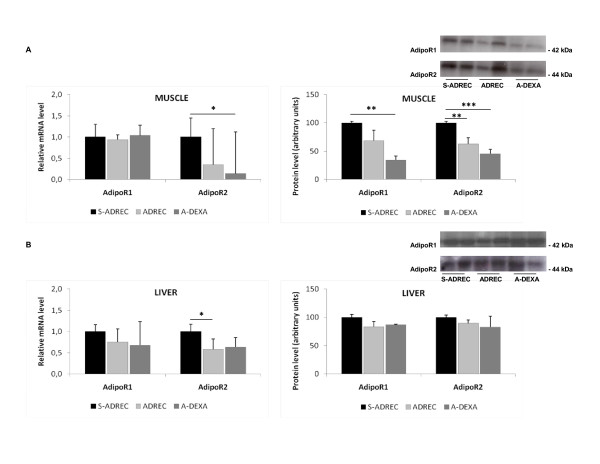
**Effects of adrenalectomy (ADREC) and adrenalectomy with dexamethasone treatment (A-DEXA; 0.2 mg/100 g, twice per day) on gene expression of the adiponectin receptors adipoR1 and adipoR2 gene in (A) muscle and (B) liver**. Data are expressed as the mean ± SEM. Values were normalized to HPRT with n = 5-9 per group. A one-way ANOVA followed by a Tukey post-hoc test was used to determine significant differences between groups (*p < 0.05, **p < 0.01, ***p < 0.005, effect due to surgery). S-ADREC, sham adrenalectomy.

We did not find any significant alterations in the mRNA levels of adipoR1 and adipoR2 in any adipose tissue depots analyzed; however, in the SUB adipose tissue of the ADREC group, the protein expression levels of adipoR1 and adipoR2 were higher than the S-ADREC or A-DEXA groups. In the EPI adipose tissue, the A-DEXA group had 66% less adipoR1 protein than the S-ADREC group (Figure 5). In the gastrocnemius muscle, the dexamethasone therapy (A-DEXA) group had a 7-fold reduction in the expression of the adipoR2 gene, and no changes were observed in adipoR1 mRNA abundance between groups. The protein expression levels of both adipoR1 and adipoR2 were reduced (55% and 65%, respectively) in the A-DEXA group compared to the S-ADREC group; a similar finding was observed with protein expression of adipoR2 in muscle (Figure 6).

Adrenalectomy reduced hepatic adipoR2 gene expression approximately 2-fold, whereas it did not affect adipoR1 gene expression or alter protein levels of either receptor (Figure 6).

## Discussion

The present study evaluated the effects of a high-fat diet on the expression of adiponectin and adiponectin receptors in rats. These rats did not become obese during the study because the percentage of fat in their diet and the period of diet treatment were not great enough to alter the body weight gain or the carcass lipid content compared to the group fed normal chow. Therefore, we can be sure that our results were restricted to the effects of the diet and were not due to metabolism changes induced by obesity.

Some studies show that adiponectin plays a role in the reduction of insulin sensitivity caused by high-fat diet [39,40]. Our results showed that 21 days of a high-fat diet containing 20% fat was not enough to modify insulin serum concentrations although there was an increase in glycemia. We demonstrated that hyperglycemia promoted by the high-fat diet was accompanied by a reduction in adiponectin gene expression in the SUB adipose tissue, but did not lead to a decrease in serum adiponectin levels, suggesting that there is a compensatory effect of the other fat depots on serum adiponectin levels. These results demonstrate the complexity of post-transcriptional mechanisms regulating adiponectin mRNA. The isolated effect of the high-fat diet on the SUB adipose tissue may be due to the fact that adiponectin gene and protein levels are higher in SUB adipose tissue than in other adipose depots [41,42].

Previous studies have suggested that increased insulin levels due to a high-fat diet may affect expression of the adiponectin receptors [18,43,44]. This insulin regulatory effect on adiponectin receptor expression is controversial because it has been shown that insulin represses the expression of both adiponectin receptors in muscle and liver [43], that adipoR1, but not adipoR2, is repressed by insulin [45], that there is no difference in adipoR1 expression in the muscles of human diabetics compared to non-diabetics [46,47] and, similar to our results, that insulin increases adipoR1 expression in muscle [48,49]. In our study, we also demonstrated that there was a decrease in adipoR1 protein expression in the RET adipose tissue. However, because the insulin levels in our HF group and C group were similar, we can assume that the alterations in the expression of adiponectin receptors were not due to insulin. Free fatty acid levels have also been found to upregulate adiponectin receptor expression [46].

Some previous studies have shown that diet-induced obesity is associated with insulin resistance in liver and muscle, but not in adipose tissue [50,51]. However, in the present study, we demonstrated that adipoR1 protein expression increased in muscle and adipoR2 gene expression increased in muscle and liver after 3 weeks of HF diet, suggesting that this may be a compensatory mechanism that acts to protect an organism from insulin resistance induced by HF diet because it has been well documented that adiponectin expression in muscle promotes an increase in insulin sensitivity [52,53]. Similar results were found in a study by Barnea et al [49] in which HF diet enhanced adipoR1 and adipoR2 expression in the muscles of mice fed with a 22% soybean fat diet for 4 months.

Peng et al [54] have proposed that adiponectin signaling may be a crosslink between HF diet, hepatic inflammation and nonalcoholic fatty liver disease. In fact, they found that HF diet-induced liver steatosis was associated with a reduction in serum adiponectin levels and downregulation of adipoR2 expression in the liver. We found that adipoR2 gene expression was increased in the livers of the HF group of rats. A similar result was found by Bullen et al [18] in mice fed a high-fat diet. These results indicate that other factors may play a role in the regulation of the adiponectin receptors in the liver.

The stimulation of adrenal glucocorticoid secretion by HF diet has been described in animals and humans [55,56]. Accordingly, Wohlers et al [21] showed that feeding rats for 7 weeks with a HF diet that was supplemented with soybean oil or fish oil (15% fat) resulted in increased serum corticosterone levels compared to those in rats fed a control diet (4% fat). Our animals fed with a soybean oil HF diet (15% fat) for only 3 weeks did not increase the serum levels of corticosterone compared to those of the control group. After 3 weeks of HF diet treatment, Drake et al [57] observed an increase in glucocorticoid clearance by hepatic A-ring reductase. The authors of this study suggested that this mechanism could protect against the metabolic complications of obesity induced by HF diet.

In HF diet-treated rats, 72 h after adrenalectomy, glycemia and insulin levels were similar to the S-ADREC group. However, glucocorticoid therapy via dexamethasone treatment in adrenalectomized rats promoted hyperglycemia and hyperinsulinemia. This treatment also decreased adiponectin serum concentrations, which was accompanied by a decrease in adiponectin mRNA in EPI adipose tissue, demonstrating that the excess of glucocorticoids rather than the glucocorticoids themselves *per se *influence adiponectin, glucose and insulin serum concentrations. Chronic treatment with glucocorticoids may result in insulin resistance followed by hyperinsulinemia and hyperglycemia [58-60]. These alterations of insulin and glucose levels can be partially explained by a decrease in insulin-dependent glucose uptake and an increase in liver gluconeogenesis. Another potential explanation could be the decrease in adiponectin serum levels observed in the present study. Adrenalectomy may increase adiponectin gene expression in SUB adipose tissue even when combined with HF diet treatment because HF diet alone was able to reduce adiponectin gene expression. With regard to expression of the adiponectin receptors in SUB adipose tissue, adrenalectomy increased adipoR1 and adipoR2 protein levels, and the dexamethasone therapy reversed these effects. AdipoR2 protein expression in muscle was reduced by adrenalectomy, but, upon the addition of glucocorticoid therapy, the adipoR2 mRNA levels did not reach the normal mRNA levels observed in the sham group. This reduction in adipoR2 expression may be due to other hormones secreted by the adrenal glands, such as mineralocorticoids and catecholamines, and, for this reason, the treatment with dexamethasone was not able to reverse the adipoR2 reduction. Furuhashi et al [61] observed that rennin-angiotensin-aldosterone system blockade increases serum adiponectin concentrations with improvement in insulin sensitivity in patients with hypertension. To our knowledge, no direct effects of aldosterone on adiponectin and adiponectin receptors were observed despite the fact that both adiponectin receptors, adipoR1 and adipoR2, have been showed to be expressed in mouse adrenal gland and adrenocortical Y-1 cells [62] and in human adrenal cortex and aldosterone-producing adenoma tissue [63]. Few studies showed that exogenous administration of dehydroepiandrosterone (DHEA) upregulated adiponectin expression and secretion from adipose tissue of rats [64,65]. Some studies have analyzed the effects of catecholamines on the regulation of adiponectin and adiponectin receptor gene and protein expression. Catecholamines inhibit adiponectin mRNA expression in 3T3-L1 adipocytes [66] and adipoR1 and adipoR2 expression in cultured cardiomyocytes [67]. In addition, Iwen et al [68] recently showed that an increase in sympathetic nervous activity caused a decrease in plasma adiponectin levels in humans. Oana et al [10] showed that the β3-adrenoceptor agonist CL-316 increased plasma adiponectin levels and the expression of adiponectin mRNA in the EPI white adipose tissue of *db/db *mice. Another study by Fu et al [69] demonstrated that treatment of 3T3-L1 adipocytes with the β-adrenoceptor agonist isoproterenol increased expression of the adipoR2, but not the adipoR1 receptor. These results are in accordance with the findings of the present study related to the effects observed in the A-DEXA group. However, more studies are needed to better understand the effects of catecholamines on the regulation of the adiponectin system because some diseases associated with obesity are related to sympathetic nervous system activity.

## Conclusion

Taken together, these results demonstrate that hyperglycemia due to a high-fat diet is associated with a decrease in the expression of adiponectin receptors in the gastrocnemius muscle. Also, our results suggest that adrenalin has a regulatory role in adipoR2 protein expression in skeletal muscle.

## List of abbreviations

adipoR1: adiponectin receptor 1

adipoR2: adiponectin receptor 2

MUFA: monounsaturated fatty acids

PUFA: polyunsaturated fatty acids

C: control diet

HF: high-fat diet

RET: retroperitoneal adipose tissue

EPI: epididymal adipose tissue

SUB: subcutaneous adipose tissue

MUSC: gastrocnemius muscle

S-ADREC: shaw adrenalectomized

ADREC: adrenalectomized

A-DEXA: adrenalectomized followed by dexamethasone reposition

## Competing interests

The authors declare that they have no competing interests.

## Authors' contributions

CO designed the study, carried out the experiments, performed the statistical analysis and drafted the manuscript. ABMM helped to carried out the experiments. CB helped to carried out the experiments. LMO participated in the design of the study helped to carried out the experiments. EBR revised and helped to draft the manuscript. CON conceived of the study, participated in its design, coordination and helped to draft the manuscript. All authors read and approved the final manuscript.
